# mSphere of Influence: Of lipoproteins and lipopolysaccharide

**DOI:** 10.1128/msphere.00185-26

**Published:** 2026-06-11

**Authors:** Cameron S. Roberts

**Affiliations:** 1 Department of Microbiology and Immunology, University of Michigan Medical School, Ann Arbor, Michigan, USA; The University of Arizona, Tucson, Arizona, USA

**Keywords:** cell-envelope, crosstalk, protein transport, gram-negative, bacteria

## Abstract

Cameron Roberts is a bacteriologist researching protein transport systems in bacteria. In this mSphere of Influence article, he highlights two studies on the transport of lipoproteins to the cell surface and their interaction with the lipopolysaccharide machinery in two divergent diderm bacteria, *Escherichia coli* (Luo et al.) *and Borrelia burgdorferi* (He et al.). These bacteria have very different outer membrane lipoprotein and lipopolysaccharide compositions, but both appear to have a connection between the lipopolysaccharide transport machinery and lipoproteins. One study provides strong evidence for the role of the lipopolysaccharide transport machinery in lipoprotein surface localization and the other shows data supporting a direct molecular interaction between these components. These works ultimately demonstrate the importance of scientific study in diverse model organisms and demonstrate that by studying what makes one species unique reveals unexpected commonalities with others.

## COMMENTARY

Bacteria are protected from the environment by the cell envelope, which in diderm bacteria such as the Gram-negative model organism *Escherichia coli* consists of an asymmetric outer membrane, periplasm, and inner membrane. Asymmetry in the outer membrane arises from the presence of lipopolysaccharide (LPS) or lipooligosaccharide (LOS), depending on the species, that decorates the outer leaflet of the outer membrane. LPS adds extra protection from environmental stressors to bacteria like *E. coli* and the dogma in the field had for many years stated that LPS is an essential component of the outer membrane of all diderm bacteria. However, some bacteria display LOS on their outer membrane such as *Neisseria meningitidis* and *Acinetobacter baumannii* and can shed their LOS through mutations in LOS biosynthesis genes (1, 2). Still other diderm bacteria such as the spirochete *Borrelia burgdorferi* natively lack LPS/LOS completely (3). In *E. coli,* LPS is transported to the outer membrane by the Lipopolysaccharide Transport (LPT) machinery, which includes the outer membrane protein LptD.

In addition to LPS, the outer membrane of diderm bacteria contains integral outer membrane proteins that often form β-barrel structures and allow for the transport of molecules across this protective barrier. The outer membrane also contains another class of proteins called lipoproteins, which consists of proteins that are posttranslationally modified with acyl chains that enable their membrane association. In *E. coli*, lipoproteins often reside in the inner leaflet of the outer membrane, transported there by the Localization of Lipoprotein (Lol) pathway. The Lol pathway consists of multiple proteins including the outer membrane protein LolB, which is responsible for inserting lipoproteins into the inner leaflet of the outer membrane. In addition, a small subset of lipoproteins is thought to be displayed on the cell surface in *E. coli* (4). Alternatively, in the case of bacteria such as *Bacteroides thetaiotaomicron* and *B. burgdorferi*, the outer membrane has an unusually high presence of surface-localized lipoproteins, which are thought to contribute to their specific lifestyles (5, 6). Researchers have extensively studied the Lol pathway, particularly in *E. coli*, but the mechanism(s) of how lipoproteins reach the cell surface in diderm bacteria are now just being recognized along with a growing appreciation of the importance of surface lipoproteins. Specific mechanisms of surface localization of lipoproteins have been described but do not appear universal among diderm bacteria (7, 8).

Given the extensive presence and importance of lipoproteins decorating the surface of *B. burgdorferi*, this model system was a natural choice to investigate lipoprotein surface localization mechanisms. Work performed by He et al. (9) identified a homolog of the β-barrel protein LptD in this organism, a striking discovery given that *B. burgdorferi* lacks LPS and LOS. The researchers went on to demonstrate that this protein, BB0838, is essential for the viability of *B. burgdorferi* similar to LptD in *E. coli*. But instead of facilitating transport of LPS, BB0838 is responsible for the surface localization of one of the major lipoproteins, OpsA. This was determined by using CRSPR interference technology to knock down BB0838 expression and then monitoring the sensitivity of OspA to proteolysis on intact cells. Using quantitative proteomic analysis, they later expanded their approach to analyze the global surface localization of lipoproteins in *B. burgdorferi* in the presence and absence of BB0838 knock down expression. With this unbiased approach, additional BB0838-dependent surface lipoproteins were identified, presenting a compelling case for the ability of an LptD homolog to mediate surface localization of lipoproteins. The authors went on to propose that as of yet uncharacterized additional proteins encoded by *B. burgdorferi*, potentially other homologs of *E. coli* LPS transport proteins, facilitate the periplasmic transport of lipoproteins in a similar manner to LPS transport.

More recently, Luo et al. have provided strong molecular evidence for a physical interaction between LptD and lipoproteins (10). Initially, the researchers observed that the purification of either *Pseudomonas aeruginosa* or *Shigella flexneri* LptD in complex with additional Lpt components using an *E. coli* expression system resulted in the co-purification of *E. coli* lipoproteins. One of the most abundant co-purified lipoproteins was LptM, which itself is implicated in the transport of LPS (11). The researchers were able to solve the structures of the LptD containing complex from *P. aeruginosa* with and without LptM using crystallography. Through a series of elegant photocrosslinking assays performed in *E coli* and in reconstituted nanodiscs, the researchers demonstrated that the lipoprotein LptM initially interacts with members of the Lol pathway before being handed off to the Lpt machinery. This is facilitated, in part, by a direct interaction between Lpt and Lol components. Subsequent surface localization was assessed for each LptD interacting lipoprotein, including LptM, using a dot blot assay where intact and lysed *E. coli* cells were probed with specific antibodies, indicating that at least a portion of the total cellular lipoproteins could be localized to the cell surface. It should be noted that a more quantitative method could provide stronger evidence for surface localization and a subsequent structural study proposed that LptM is periplasmic (12). Regardless, the study of Luo et al. provides a potential mechanism for the direct interaction of LPS machinery and lipoproteins.

Reflecting on these and other works, it is clear that the field of microbiology is moving toward using increasingly diverse model organisms. By studying lipoprotein trafficking in *B. burgdorferi* and *E. coli*, researchers revealed an unexpected connection between lipoproteins and lipopolysaccharides. It is quite possible that a new connection between LPS and lipoprotein transport would not have readily been appreciated if researchers limited their efforts to the perennial model organism *E. coli*. It was only after recognizing the uniqueness of the *B. burgdorferi* outer membrane that researchers began to investigate the role of LptD in that organism and then appreciate a new mechanism shared with *E. coli*. There may be additional outstanding questions we can address harnessing the diversity across Gram-negative bacteria. For example, the lipoprotein trafficking protein LolB is responsible for inserting lipoproteins into the outer membrane, but LolB is itself a lipoprotein. LolB has extensively been studied in *E. coli* but is just beginning to be characterized in additional organisms. And while some bacteria harbor LolB like *E. coli*, others do not. For those lacking LolB, how do they insert lipoproteins into the outer membrane? Indeed, researchers are already working on what makes these bacteria unique and have discovered that some bacteria such as *Caulobacter vibrioides* have another lipoprotein trafficking protein that compensates for the missing LolB (13), indicating an alternative mechanism of lipoprotein outer membrane insertion. Perhaps further lipoprotein/lipopolysaccharide crosstalk could explain LolB’s chicken or egg predicament in *E. coli* and work done in non-traditional model organisms such as *C. vibrioides* could help solve this issue.
